# The Immediate Hypoalgesic Effects of Mobilization and Manipulation in Patients with Non-Specific Chronic Low Back Pain: A Cross-Over Randomized Controlled Trial

**DOI:** 10.3390/healthcare13141719

**Published:** 2025-07-17

**Authors:** Thomas Sampsonis, Stefanos Karanasios, George Gioftsos

**Affiliations:** Physiotherapy Department, School of Health and Care Sciences, University of West Attica, 12243 Aigaleo, Greece; t.sampsonis@gmail.com (T.S.); gioftsos@uniwa.gr (G.G.)

**Keywords:** low back pain, manual therapy, thrust, pain sensitivity

## Abstract

**Background/Objectives:** Manual therapy techniques, including mobilization and manipulation, are commonly used for chronic low back pain (CLBP), with clinical guidelines recommending their use. This study aimed to compare the immediate hypoalgesic effects of mobilization and manipulation in patients with non-specific CLBP, evaluating their impact on pain sensitivity and range of motion. **Methods:** A cross-over randomized controlled trial was conducted with 27 participants with non-specific CLBP. Participants received either mobilization or manipulation on two different intervention days. Outcome measures included pressure pain thresholds (PPTs) assessed with a digital algometer, pain intensity using a numeric rating scale, and lumbar range of motion (ROM) measured with a digital inclinometer. **Results:** The results indicated no statistically significant differences between mobilization and manipulation for any outcome measures (all *p* > 0.05). However, significant within-intervention improvements were observed, including pain reduction, increased PPTs, and enhanced ROM of the lower back. **Conclusions:** Our findings suggest that both mobilization and manipulation provide similar immediate benefits for patients with CLBP. The choice between these techniques should be based on therapists’ clinical reasoning and individualized risk stratification, considering the potential benefits and risks of each approach for a specific patient.

## 1. Introduction

Low back pain (LBP) is one of the most common disabling disorders worldwide, with a prevalence of 11.9% in adults [1,2]. It causes a significant social and economic burden that is driven primarily by 5% to 15% of patients who develop chronic symptoms (>3 months) [3,4]. Providing effective treatment strategies to reduce symptoms and improve function in chronic LBP (CLBP) is considered critical for patients, clinicians, and stakeholders. Current therapeutic strategies for cLBP include pharmacological and nonpharmacological treatments, such as spinal manipulative therapy (SMT), therapeutic exercises, education, or multidisciplinary rehabilitation [5].

Manipulative therapies are considered a first-line conservative treatment approach involving manipulation or mobilization techniques. Manipulation techniques apply a high-velocity, small-amplitude thrust to the spine and are often accompanied by an audible pop [6]. In contrast, mobilizations are low-velocity techniques, using a low force, and they can be performed in various parts of an available range according to the desired effect. Evidence suggests that both manual therapy techniques are effective in lowering pain, improving function, and increasing mobility of the lower back in people with CLBP [5]. Despite multiple reports about their clinical effectiveness, the research evidence for the underlying mechanisms of action—remains inconclusive [7,8].

The main underlying mechanisms of action when using SMT include biomechanical changes and neurophysiological effects on the central and peripheral nervous systems by influencing the spinal and supraspinal levels [9]. Specifically, spinal mobilization and manipulation techniques have been reported to change the processing of sensory stimuli at the level of the brain and the spinal cord by increasing the cortical activity in the periaqueductal gray region and decreasing neural excitability at the level of the dorsal horn neurons (descending inhibitory pain mechanism) [9,10,11]. In an attempt to better understand these mechanisms of action, past studies have investigated the changes in the pressure pain threshold (PPT), suggesting significant changes at local, regional, and remote sites after SMT [5,12,13,14]. However, the clinical interpretation of these findings requires caution due to the sample characteristics (asymptomatic subjects), the type of intervention, and the length of follow-up [10]. Also, despite the ongoing research in the current field, there is still controversy surrounding which technique is superior and whether clinician decision-making impacts patients’ outcomes. Current evidence suggests that both spinal mobilization and manipulation are safe and demonstrate comparable short-term effects in reducing pain for individuals with non-specific chronic low back pain (NS-CLBP). However, manipulation appears marginally more effective in some clinical outcomes. Consequently, clinicians should base their decisions on patients’ potential risks and beliefs [15,16].

Based on the authors’ best knowledge, there is no study evaluating the comparative effectiveness of lumbar spine mobilization and manipulation on pain sensitivity in patients with CLBP. Therefore, the primary aim of this randomized controlled trial (RCT) was to compare the effect of the two different manual therapy techniques on PPTs at local and remote sites, in people with non-specific CLBP. The secondary outcomes included the changes in pain intensity and range of motion (ROM) of the lumbar spine before and after the interventions.

## 2. Materials and Methods

### 2.1. Study Design

A prospective, cross-over randomized controlled design was implemented in a physiotherapy clinic in Attica, Greece. Participants were recruited from November 2023 to February 2024 via advertisements at the University of West Attica and from orthopedic consultants’ referrals. One physiotherapist (TS) with nine years of clinical experience and expertise in manual therapy delivered both interventions. The trial was prospectively registered in clinicaltrials.gov (NCT06757400). The study protocol was approved by The Research Ethics Committee of the University of West Attica (ID: 65273/7 July 2023).

### 2.2. Eligibility Criteria

**Figure 1:**
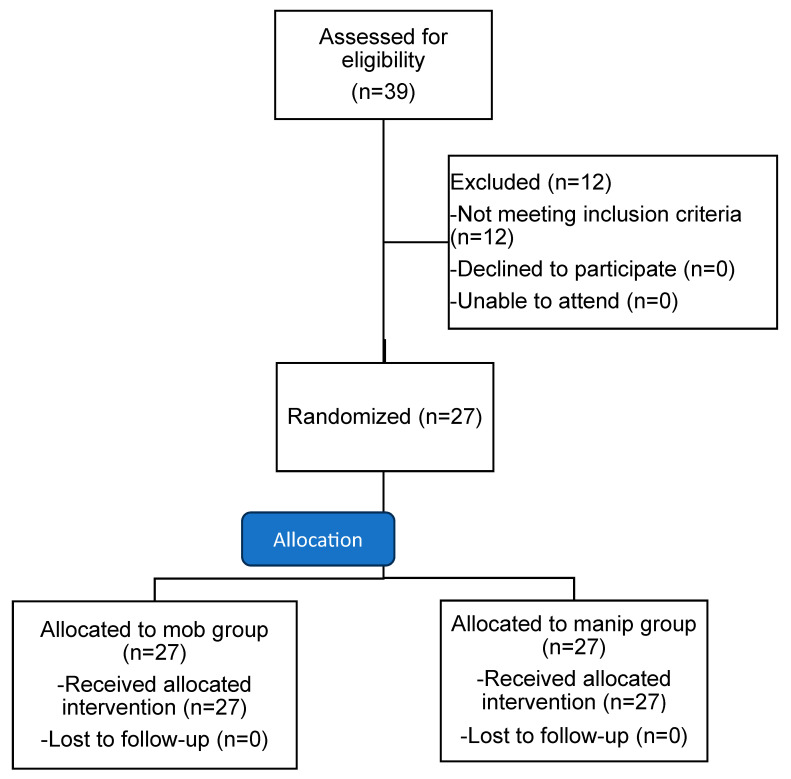
Consort statement flow diagram. Abbreviations: mob, mobilization; manip, manipulation.

Participants were adults aged 18–60 with non-specific CLBP and symptoms persisting for more than 12 weeks. Non-specific CLBP was defined as a mechanical pain located in the lower back, without a specific nociceptive source identified during the medical assessment [17]. The exclusion criteria included the following: spinal fractures, intervertebral disc herniation with neurological deficit, medical diagnosis of spondylolisthesis, degenerative joint diseases (e.g., osteoarthritis, spondylarthropathy), osteoporosis, infection (e.g., osteomyelitis), hippurid syndrome, tumors of the spinal column or spinal cord, spinal injury, and previous spinal surgery. These criteria follow guidelines from the World Health Organization for the contraindications of spinal manipulations [18] and the European guidelines for the diagnosis of non-specific CLBP [19]. The participants also needed to lack symptoms of radicular pathology, such as unilateral leg pain greater than lumbar pain, and radiating leg pain. Also, the straight leg raise test should not increase pain intensity. Red flags such as chest pain, cancer history, steroid use, unexplained weight loss, and general malaise were screened out [19,20]. The initial assessment for eligibility was conducted by a physiotherapist with 20 years of experience (SK). We adhered to the Consolidated Standards of Reporting Trials (CONSORT) recommendations for designing and reporting the present study (Figure 1).

### 2.3. Randomization and Masking

An independent researcher performed randomization of the two interventions in two sequences (mobilization and manipulation) using online randomization software (https://www.randomizer.org/) with a 1:1 allocation ratio. Another independent researcher (AT) was responsible for contacting patients and allocating them to groups according to each condition using consecutively numbered, sealed, opaque envelopes. The participants were instructed not to talk with the assessor or any other participant about the type of intervention they received.

Before each intervention, an independent assessor (PCT) collected demographic characteristics (sex, age, height, weight, employment type, and symptoms duration), pain intensity, lumbar ROM, and PPTs. Then, the subjects visited another room where the intervention was delivered. Due to the study design (cross-over trial), the physiotherapist (TS) delivering both interventions was not blinded to the group allocation; however, he was instructed to provide equal motivation and treatment for both groups.

### 2.4. Interventions

**Figure 2:**
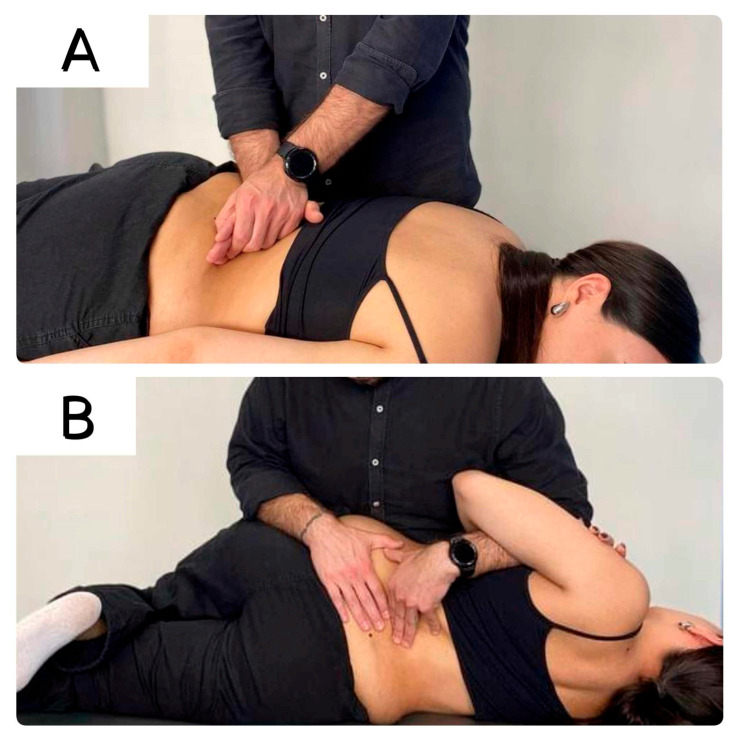
Manual therapy interventions: (**A**) The therapist applied a postero-anterior mobilization on the most hypomobile vertebral level (Grade IV Maitland’s mobilization); (**B**) The therapist applied a side-lying rotational lumbar thrust manipulation at the most hypomobile lumbar segment, identified in the initial assessment [22,23].

The participants were positioned to lie in a prone position and the therapist (TS), applied a clinical evaluation of the lumbar vertebrae stiffness (normal, hyperkinetic, or hypokinetic) by applying a gentle mobilization to the spinous processes of the L1 to L5 vertebrae from posterior to anterior [21]. Then, each participant received either a mobilization or manipulation technique at the most hypokinetic segment (Figure 2A,B). The two interventions (i.e., lumbar spine mobilization or manipulation) were randomly applied on two different days, one week apart. The participants were required to refrain from taking anti-inflammatory or analgesic medication on the days of the interventions.

The side on which the patient was positioned for the intervention (mobilization or manipulation) was not standardized across participants but was determined clinically based on the location of reported pain or observed dysfunction during the initial assessment. Although this individualized approach may introduce a degree of variability, current evidence suggests that side-specific effects are likely minimal in symptomatic populations [24]. Wong et al. reported no significant differences in pain modulation between left- and right-sided cervical spinal manipulations in individuals with chronic neck pain [25]. Similarly, Colloca et al. demonstrated comparable bilateral neurophysiological responses following unilateral lumbar spinal manipulation [26]. A systematic review by Lystad et al. further concluded that manipulation laterality does not exhibit a consistent influence on clinical outcomes [27]. In line with these findings, Currie et al. observed no significant electromyographic differences during spinal manipulation in asymptomatic subjects [28].

### 2.5. Outcome Measures

To measure PPTs, a digital algometer (Baoshishan ZP-1000 N 20/22806, China) was used following the same sequence of measurements pre- and post-interventions [4]. The body sites of measurements included the following: 5 cm lateral to the L5 spinous process, bilaterally [29,30]; the L5 spinous process [12]; the upper trapezius muscle (midway between the C7 spinous process and acromion), bilaterally [31]; and the tibialis anterior muscle (5 cm lateral to the tibial tubercle), bilaterally [29,32]. The interrater reliability of the current method has been found excellent for the tibialis anterior muscle (ICC = 0.91, 95% CI 0.31–0.95) and lumbar muscles (ICC = 0.82, 95% CI 0.65–0.97) in a chronic low back pain population, when measured over 48 h [33]. Threshold limits were measured in prone and sitting positions with three consecutive measurements taken at each point at 1 min intervals. Pressure with the algometer was applied at a rate of 30 kPa/second using a visual indicator on the algometer. The participants were instructed to say ‘now’ at the moment they felt the sensation of pain. The average of three measurements for each site was recorded and the same procedure was carried out before and after each intervention [30,34].

For subjective pain assessment, the participants completed a self-reported numerical pain rating scale (NPRS) ranging from 0 to 10, with “0” indicating no pain and “10” indicating the maximum possible pain, covering pain intensity over the previous week. This scale was administered pre- and post-intervention, showing predictive validity in chronic low back pain patients [35,36].

Lumbar ROM was measured in sagittal (flexion and extension) and frontal planes of motion (right and left lateral flexion) using an electronic goniometer. The participants stood with feet shoulder-width apart, starting from a neutral position and returning there after each movement. The average of three repetitions for each movement (flexion, extension, and right and left lateral bending) was used for data analysis [37]. The measurements were taken before and immediately after each intervention. Before using the electronic goniometer, familiarization tests were conducted to determine intra-rater reliability, which was found excellent for flexion (ICC: 0.99), extension (ICC: 0.95), right lateral flexion (ICC: 0.93), and left lateral flexion (ICC: 0.96) [38].

### 2.6. Sample Size

Based on previous studies that have evaluated the immediate effects of manipulative therapy in cLBP patients, a sample size calculation was conducted (G*Power 3.1) by using PPTs as a primary outcome measure [4,39]. A sample size of 27 individuals in each group was estimated to be sufficient to detect an effect size of 0.5 (power 0.80, 2-sided significance level 0.05) on the PPTs. PPTs, a reliable tool for assessing localized pain sensitivity, demonstrate high intra- and inter-rater reliability, with common testing sites including the upper trapezius and tibialis anterior. Clinicians should note that a minimal clinically important difference (MCID) of 1.63 kg/cm^2^ (160 kPa) is recommended for interpreting PPT changes in clinical practice [40,41,42].

### 2.7. Statistical Analysis

Data normality was visually assessed using a Q–Q plot and statistically via the Shapiro–Wilk test. Descriptive statistics were used for baseline characteristics, presented as means and standard deviations (SD) with 95% confidence intervals (CIs). A mixed-effects model analyzed the differences between the two groups, with a participant as a random effect and two measurement time points (pre- and post-intervention). Fixed effects included group, time, and group-by-time interactions, adjusted for sex, age, BMI, and symptom duration. Post hoc tests with Bonferroni adjustments were performed where significant main effects or interactions were found. For secondary outcome measures, we also applied Bonferroni adjustments to account for multiple comparisons. To account for potential order effects, mixed-effects models could be used, with ‘treatment order’ included as a fixed effect and ‘participant’ modeled as a random effect, thereby accounting for within-subject variability.

## 3. Results

From November 2023 to February 2024, 39 individuals with cLBP expressed interest and consented to participate in the study. Of these, five were excluded due to age, four due to radiating pain in the lower limbs, and three because of comorbid conditions, including rheumatoid arthritis and osteoporosis. Eventually, 27 participants met all the inclusion criteria, provided written informed consent, and completed the study, with no dropouts (Figure 1). The mean (±SD) age of the participants was 38.4 (±11.2) years, and the mean duration (±SD) of symptoms was 56.1 (±47.7) months. The participants’ demographic characteristics are shown in Table 1.

### 3.1. Between-Group Differences

There were non-statistically significant differences (*p* > 0.05) between mobilization and manipulation techniques in PPTs at the spinous process of the L5 (0.15, 95%CI: −0.94 to 1.23 *p* = 0.79), left (0.16, 95%CI: −0.76 to 1.07, *p* = 0.73), and right (−0.39, 95%CI: −1.17 to 0.4, *p* = 0.33) paravertebral area of the L5 spinous process left (0.11, 95%CI: −0.5 to 0.72, *p* = 0.73) and right trapezius (0.36, 95%CI: −0.22 to 0.93, *p* = 0.22), and the left (0.33, 95%CI: −0.66 to 1.32, *p* = 0.51) and right tibialis anterior muscles (−0.08, 95%CI: −1.04 to 0.87, *p* = 0.86) (Table 2).

There was a non-significant difference between the two interventions in pain intensity measured with NPRS (0.19, 95%CI: −0.48 to 0.85; *p* = 0.58) immediately after each treatment (Table 3). Non-significant differences were found between mobilization and manipulation groups post-treatment in lumbar ROM for the flexion (−1.59, 95%CI: −4.26 to 1.08; *p* = 0.24), extension (0.15, 95%CI: −1.82 to 2.11, *p* = 0.88), right (−0.13, 95%CI: −1.95 to 1.68; *p* = 0.88), and left lateral flexion (−0.34, 95%CI: −2.3 to 1.61; *p* = 0.73) (Table 3).

### 3.2. Within-Group Differences

Statistically significant within-group changes were found between pre- and post-intervention in the mobilization group at the left (−1.19, 95%CI: −2.11 to −0.27; *p* = 0.01) and right paravertebral area of the L5 spinous process (−0.95, 95%CI: −1.74 to −0.17; *p* = 0.018); the left (−0.83, 95%CI: −1.43 to −0.22; *p* = 0.009) and right trapezius (−1.18, 95%CI: −1.76 to −0.61; *p* = 0.0001). Similarly, significant differences in PPTs were observed in the manipulation group at the left (−1.38, 95%CI: −2.29 to −0.46; *p* = 0.004) and right (−1.24, 95%CI: −2.03 to −0.46; *p* = 0.002) paraspinal area of the L5 spinous process, and the left (−0.9, 95%CI: −1.51 to −0.29; *p* = 0.004) and right trapezius (−0.99, 95%CI: −1.58 to −0.42; *p* = 0001). A significant difference between pre- and post-intervention was also observed in pain intensity in the mobilization (2.15, 95%CI: 1.48 to 2.81; *p* < 0.0001) and manipulation (2.15, 95%CI: 1.48 to 2.81; *p* < 0.001) group. There were non-significant within-group changes in the PPTs at the rest sites of measurements and the lumbar ROM in both groups between pre- and post-interventions (Table 4). The results showed that there were no statistically significant differences (*p* > 0.05) with respect to the time factor for all outcome measures (pain intensity (*p* = 0.27); PPTS for L5 (*p* = 0.76), left (*p* = 0.78), and right (*p* = 0.28) paravertebral area of the L5 spinous process, the left (*p* = 0.12) and right trapezius (*p* = 0.12), the left (*p* = 0.7) and right tibialis anterior muscles (*p* = 0.92), and in lumbar ROM for the flexion (*p* = 0.4), extension (*p* = 0.8), right (*p* = 0.1), and left lateral flexion (*p* = 0.96)), (see Supplementary Materials).

## 4. Discussion

Our study results suggest that despite the significant reduction in mechanical pain intensity and subjective pain intensity for both mobilization and manipulation groups when comparing pre- and post-intervention time periods, there were no statistically significant differences in the between-groups results. Also, there were no differences between the interventions in lumbar ROM at all planes of movement (i.e., flexion, extension, right lateral flexion, and left lateral flexion) immediately after treatment.

Based on the authors’ knowledge, this is the first study to evaluate the changes in mechanical pain sensitivity of manipulation compared to mobilization in patients with CLBP. The results of the present study align with the findings of [43] who reported no significant differences in PPTs between mobilization and manipulation in the thoracic spine among a healthy population. Although we identified a significant hypoalgesic effect immediately post-intervention for both groups, our findings differ from the available evidence. According to another RCT that evaluated changes in cervical PPTs between mobilization and manipulation in people with chronic mechanical neck pain, there were no significant interaction effects between time and intervention [44]. However, the absence of significant reductions in pain sensitivity in the previous study could be because the participants were not asked to refrain from taking medication on the day of the intervention. In line with this, a recent systematic review and meta-analysis suggested no significant immediate effects on PPTs after spinal manipulation techniques [45]. However, the results of this review should be interpreted with caution due to the increased heterogeneity of the study population, which included both patients with chronic musculoskeletal disorders and pain-free individuals [45]. The discrepancies found between the studies may be attributed to methodological differences, such as the use of pain as an outcome measure and its broad assessment range, as well as the wide age spectrum of the sample.

According to our findings, both interventions significantly reduced subjective pain intensity at rest. In agreement with these results, previous research has suggested no statistically significant differences between the two interventions in terms of pain intensity [16,46]. Manual therapy is widely used to manage low back pain [7], but its effectiveness depends heavily on individual patient factors. Therefore, clinical prediction rules and individualized treatments are crucial for optimizing manual therapy outcomes [9,21]. Understanding the complex interplay of the biomechanical and neurophysiological mechanisms of manual therapy is essential to interpreting the present findings. Biomechanically, manual therapy is thought to directly stimulate tissues, potentially improving spinal biomechanics and circulation [7]. Neurophysiologically, it is considered to modulate nociceptive input at the peripheral, spinal, and supraspinal levels, affecting pain perception and potentially inducing reflexive muscle relaxation [9]. The hypoalgesic effects of spinal manipulation (SM) appear to be mediated through three distinct but potentially interacting neurophysiological mechanisms. First, segmental mechanisms involving modulation of dorsal horn neuronal activity may lead to localized inhibition of nociceptive processing. Second, SM may activate descending pain modulatory pathways originating in brainstem structures such as the periaqueductal gray and rostroventromedial medulla, which exert inhibitory control over spinal nociceptive transmission. Third, supraspinal mechanisms involving higher cortical centers may contribute to pain modulation through altered sensory processing and cognitive-evaluative aspects of pain perception. This multilevel neuromodulatory model integrates contemporary understanding of manual therapy’s neurobiological effects with the established pain physiology framework [10,47]. While immediate effects are apparent, a purely biomechanical explanation is insufficient, and the potential influence of the placebo effect should be considered. Our results demonstrate clinically significant pain relief in most participants, with large effect sizes for both the mobilization (Cohen’s *d* = 0.97) and manipulation (Cohen’s *d* = 1.1) groups. However, the absence of a placebo control group limits our ability to fully isolate the specific effects of the interventions from the influence of treatment expectations, a factor which warrants further investigation [48]. Our findings may have significant clinical implications, given that patients with LBP view immediate pain relief as a positive treatment outcome [49]. Both intervention groups achieved an average pain reduction exceeding 20%, surpassing the minimum clinically important difference for patients with CLBP receiving physiotherapy [50]. Importantly, a larger percentage of participants in both groups reached this threshold between pre- and post-intervention. The absence of reported adverse effects in either group suggests that both spinal mobilization and manipulation are safe and effective options for providing immediate pain relief in patients with chronic LBP.

### Limitations and Future Research

The present study should be viewed in light of certain limitations. First, the short-term follow-up prevents conclusions about the long-term treatment effects. Second, the nature of the interventions limited the blinding of the therapists, potentially introducing a high risk of performance bias. Third, the absence of a sham or no-treatment control group represents a significant limitation. Given the moderate effect sizes observed, this design gap may hinder the ability to distinguish between contextual factors (e.g., therapeutic touch and patient expectancy) and the specific effects of mobilization or manipulation. Furthermore, this study did not collect data on certain potentially influential factors, such as participants’ handedness (right- or left-handed), the laterality of pain (unilateral or bilateral), or their preferred lying side (painful vs. asymptomatic side). Future research would benefit from including these variables to enhance the comprehensiveness and clinical applicability of the findings. Future research should address these limitations by incorporating longer-term follow-up, employing blinding techniques where feasible, and including a placebo control group. Furthermore, evaluating multiple outcome measures, such as cold and heat pain thresholds and relevant biomarkers, would enhance the robustness of future studies.

## 5. Conclusions

This study found no significant differences between mobilization and manipulation in pain sensitivity, pain intensity, or lumbar spine range of motion. Both techniques effectively reduced PPTs at all body sites and subjective pain intensity at rest. This suggests that therapists may choose between these treatment interventions based on individual patient characteristics and clinical judgment, weighing the potential benefits against the risks and benefits ratio associated with each approach. Future research incorporating a placebo control group and longer-term follow-up is necessary to fully understand the long-term effects of mobilization and manipulation in patients with CLBP.

## Figures and Tables

**Table 1:** Participants’ baseline characteristics.

Characteristics	(Ν = 27)
Men (%)	15 (60%)
Women (%)	12 (40%)
Age (years)	38.4 ± 11.2
Height (meters)	1.74 ± 0.1
Weight (Kg)	84.4 ± 17.0
BMI	27.9 ± 5.4
Symptom duration (months)	56.1 ± 47.7

Abbreviations: SD, standard deviation; BMI, body mass index.

**Table 2:** Between-group differences of mean values of pressure pain thresholds (kg/cm^2^).

	Mobilization *	Manipulation *	Between-Group Differences †	*p*-Value	Cohen’s d
L5					
Pre-	9.85 (2.90)	9.36 (2.90)	-	-	-
Post-	10.83 (2.97)	10.68 (3.06)	0.15 (−0.94 to 1.23)	0.79 ^¶^	0.05
L5 Left paravertebral area					
Pre-	9.89 (2.70)	9.55 (2.50)	-	-	-
Post-	11.09 (2.90)	10.93 (2.70)	0.16 (−0.76 to 1.07)	0.73 ^¶^	0.09
L5 Right paravertebral area					
Pre-	9.64 (2.30)	9.74 (2.40)	-	-	-
Post-	10.60 (2.70)	10.98 (3.00)	−0.39 (−1.17 to 0.4)	0.33 ^¶^	0.13
Left trapezius					
Pre-	7.62 (2.40)	7.44 (2.60)	-	-	-
Post-	8.44 (2.40)	8.34 (2.30)	0.11 (−0.5 to 0.72)	0.73 ^¶^	0.04
Right trapezius					
Pre-	7.57 (2.40)	7.40 (2.40)	-	-	-
Post-	8.76 (2.50)	8.40 (2.40)	0.36 (−0.22 to 0.93)	0.22 ^¶^	0.15
Left tibialis anterior					
Pre-	11.87 (3.60)	11.83 (3.50)	-	-	-
Post-	12.50 (3.90)	12.17 (3.70)	0.33 (−0.66 to 1.32)	0.51 ^¶^	0.09
Right tibialis anterior					
Pre-	12.63 (3.60)	12.75 (3.90)	-	-	-
Post-	13.31 (3.90)	13.39 (4.10)	−0.08 (−1.04 to 0.87)	0.86 ^¶^	0.27

* Values are means ± SD in parentheses; † Values in parentheses are 95% confidence intervals; ^¶^ Adjustments were performed for post hoc multiple comparisons (Bonferroni). Abbreviations: SD, standard deviation; PPTs: pressure pain thresholds; Pre, before; Post, after.

**Table 3:** Between-group differences of mean values of pain intensity and lumbar range of motion.

	Mobilization *	Manipulation *	Between-Group Differences †	*p*-Value	Cohen’s d
Pain intensity (0–10)					
Pre-	2.56 (2.0)	2.59 (1.36)	-	-	-
Post-	0.63 (1.36)	0.44 (0.93)	0.19 (−0.48 to 0.85)	0.58 ^¶^	0.16
ROM (°)					
Flexion					
Pre-	68.16 (8.40)	69.57 (9.20)	-	-	-
Post-	70.67 (8.70)	72.27 (8.50)	−1.59 (−4.26 to 1.08)	0.24 ^¶^	0.19
Extension					
Pre-	14.98 (8.20)	14.29 (7.80)	-	-	-
Post-	16.01 (8.60)	15.86 (6.70)	0.15 (−1.82 to 2.11)	0.88 ^¶^	0.02
Right side flexion					
Pre-	20.54 (5.20)	20.22 (5.80)	-	-	-
Post-	21.09 (5.20)	21.23 (6.40)	−0.13 (−1.95 to 1.68)	0.88 ^¶^	0.02
Left side flexion					
Pre-	22.12 (5.20)	22.62 (5.10)	-	-	-
Post-	23.18 (5.60)	23.52 (6.00)	−0.34 (−2.3 to 1.61)	0.73 ^¶^	0.06

* Values are means ± SD in parentheses; † Values in parentheses are 95% confidence intervals; ^¶^ Adjustments were performed for post hoc multiple comparisons (Bonferroni). Abbreviations: SD, standard deviation; ROM, range of motion; Pre, before; Post, after; (°), Degrees (angle).

**Table 4:** Within-group differences (pre-post) of baseline-adjusted values of pressure pain thresholds.

	Mobilization Mean (95%CI), *p*-Value	Manipulation Mean (95%CI), *p*-Value
PPTs (kg/cm^2^)		
L5	−0.98 (−2.57 to 0.62), *p* = 0.23, d = 0.33	−1.33 (−2.92 to 0.27), *p* = 0.1, d = 0.54
L5 Left paravertebral area	−1.19 (−2.11 to −0.27), *p* = 0.01, d = 0.43	−1.38 (−2.29 to −0.46), *p* = 0.004, d = 0.53
L5 Right paravertebral area	−0.95 (−1.74 to −0.17), *p* = 0.018, d = 0.38	−1.24 (−2.03 to −0.46), *p* = 0.002, d = 0.46
Left trapezius	−0.83 (−1.43 to −0.22), *p* = 0.009, d = 0.34	−0.9 (−1.51 to −0.29), *p* = 0.004, d = 0.37
Right trapezius	−1.18 (−1.76 to −0.61), *p* = 0.001, d = 0.49	−0.99 (−1.58 to −0.42), *p* = 0.001, d = 0.42
Left tibialis anterior	−0.63 (−1.62 to 0.36), *p* = 0.21,^,^ d = 0.17	−0.34 (−1.33 to 0.65), *p* = 0.49, d = 0.1
Right tibialis anterior	−0.67 (−1.63 to 0.28), *p* = 0.16, d = 0.19	−0.64 (−1.59 to 0.31), *p* = 0.18, d = 0.16
Pain intensity		
NPRS	1.93 (1.26 to 2.6), *p* < 0.0001, d= 0.97	2.15 (1.48 to 2.81), *p* < 0.0001, d = 1.1
ROM (°)		
Flexion	−2.51 (−5.19 to 0.16), *p* = 0.07, d = 0.3	−2.7 (−5.37 to −0.03), *p* = 0.05, d = 0.31
Extension	−1.03 (−3 to 0.93), *p* = 0.3, d = 0.12	−1.58 (−3.54 to 0.38), *p* = 0.11, d = 0.22
Right side flexion	−0.56 (−2.37 to 1.26), *p* = 0.54, d = 0.11	−1.01 (−2.82 to 0.81), *p* = 0.27, d = 0.17
Left side flexion	−1.06 (−3.01 to 0.9), *p*= 0.28, d = 0.2	−0.9 (−2.85 to 1.06), *p* = 0.36, d = 0.16

Note: Adjustments were performed for post hoc multiple comparisons (Bonferroni). Abbreviations: PPTs, pressure pain thresholds; NPRS, numeric pain rating scale; ROM, range of motion; (°), Degrees (angle).

## Data Availability

Data are available upon request.

## Supplementary Materials

The following supporting information can be downloaded at: https://www.mdpi.com/article/10.3390/healthcare13141719/s1.
