# Supplementary material for: The Immediate Hypoalgesic Effects of Mobilization and Manipulation in Patients with Non-Specific Chronic Low Back Pain: A Cross-Over Randomized Controlled Trial
Source: Healthcare (Basel). 2025 Jul 17;13(14):1719. doi: 10.3390/healthcare13141719 (PMC12294813; doi:10.3390/healthcare13141719)
Supplement: Supplementary file 1 [file healthcare-13-01719-s001.zip › healthcare-3649072-supplementary.pdf]

Table S1. Effect of Treatment Order on Outcomes: Fixed Effect Model Analysis

	**Order**
**PPTs (kg/cm2)**	
L5	p=0.76
L5 Left paravertebral area	p=0.78
L5 Right paravertebral area	p=0.28
Left trapezius	p=0.12
Right trapezius	p=0.12
Left tibialis anterior	p=0.7
Right tibialis anterior	p=0.92
**Pain intensity**	
NPRS	p=0.27
**ROM (º)**	
Flexion	p=0.4
Extension	P=0.8
Right side flexion	p=0.1
Left side flexion	p=0,96
